# OA07 What is effective pain management: an illustrative case report?

**DOI:** 10.1093/rap/rkac066.007

**Published:** 2022-09-28

**Authors:** Elsje de Villiers, Nicholas Shenker

**Affiliations:** Department of Physiotherapy, Cambridge University Hospitals NHS Foundation Trust; Department of Rheumatology, Cambridge University Hospitals NHS Foundation Trust; Division of Medicine, University of Cambridge

## Abstract

**Introduction/Background:**

Pain is a sensory and emotional experience perceived in terms of tissue damage and expressed in terms of altered behaviour. Chronic, medically unexplained pain is complex, disabling and common. It is important to rule out medical or modifiable causes, but most tests will not result in significant changes in management nor prognosis.

Attempting rehabilitation before the patient narrative has been fully explained usually leads to a lack of progress. Sleep deprivation is important to acknowledge and restorative sleep has analgesic and fatigue-improving properties. Prior mood disturbances may alter management, particularly if bereavement or post-traumatic memories are present. Adverse childhood events occur more frequently than expected in this population. Somatisation and personality disorders will need expert evaluation and management. Significant anxiety and depression should be managed independent of pain report.

Physical rehabilitation is effective in restoring function which can lead to a recovery of identity and workplace productivity. Altering health beliefs and cognition can be important as part of this process. Exercise prescriptions and access to support during this process are also important. Analgesic medications usually have little to offer other than side effects, but can be used to bridge difficult times, particularly if patient expectations align with their use. Strong opioids should not be started, unless no other options are available, and always with a time- and dose-limit, preferably less than 50mg daily of morphine equivalent for less than 3 months.

We present the case of a young gentleman of working age with chronic back pain who underwent effective pain management to restore good function including a full return to work, improvement in his mood, sleep, physical function and quality of life.

**Description/Method:**

43 year old manager working as a Risk Manager developed back pain without trauma that affected all three segments. There was no morning stiffness, night pain nor gelling history. His pain was worse on exercise and when standing. The pain was intrusive and affecting his quality of life, to the extent that he could not attend work. Time away from the workplace was adding stress and affecting his mood and, intermittently, his sleep. There was no history of night sweats, weight loss, loss of appetite, significant leg pain nor numbness in his feet nor saddle area. There was no loss of control in his bowel nor urine habit.

His blood tests (FBC, LFT, Creat, ESR, CRP, vitamin D, ESR, CRP) were normal. An MRI scan of his whole spine showed minor degenerative disc changes but no significant bone changes nor neural compression.

His medications were sertraline 50mg once daily, meloxicam 7.5mg once daily and omeprazole 20mg once daily.

The pain management programme teaches self-management approaches to patients experiencing chronic pain. It is delivered by a physiotherapist, an occupational therapist, a nurse and a clinical psychologist. Its aim is to help patients to maintain or increase their physical functioning and to regain their sense of control in the context of their life. In turn, patients may improve in their well-being and their quality of life, despite their chronic pain. The programme runs for twelve days with patients attending as out-patients, four days a week for three weeks. Reviews take place at one month, six months and one year following this.

**Discussion/Results:**

Table 1 outlines the improvement in his outcomes. Self-efficacy scores rose first with associated drops in catastrophisation and kinesiophobia. Anxiety and depression scores also dropped after the first 6 weeks. Pain significantly reduced along with improvements in quality of life and return to full employment 6 months later.

**Key learning points/Conclusion:**

Patients with chronic pain have significant disability, poor quality of life and are not economically productive. Pain management programmes are well evidenced to restore function and improve quality of life. Pain scores typically drop later and full employment can be regained, thus suggesting a cost-effectiveness of such programmes for a population.

**OA07 Table 1:** Patient reported questionnaire outcome measures

	02/11/2020	19/11/2020	15/12/2020	07/07/2021	31/12/2021
Time	Baseline	2 weeks	6 weeks	7 months	13 months
Back pain /10	9	7	7	7	4
Leg pain /10	2	1	0	1	0
Leg numbness /10	1	0	0	2	0
Leg tingling/10	1	0	0	1	0
RMDQ	14	9	8	4	3
EQ-5D-5L / 25	18	18	16	12	10
HAD – A / 21	9	7	6	4	5
HAD – D / 21	8	8	5	3	4
PSEQ / 60	22	38	30	46	47
PCS /39	29	20	15	13	11
TSK / 68	38	31	32	26	30
Employment	Benefits	Benefits	Benefits	Full time	Full time

*RMDQ: Roland Morris Disability Questionnaire* – lowest scores are healthiest

*EQ-5D-5L: EuroQoL’s 5 Dimensions with 5 Levels* – lowest scores are healthiest

*HAD – A: Hospital Anxiety and Depression – Anxiety Scale* – lowest scores are healthiest

*HAD – D: Hospital Anxiety and Depression – Depression Scale* – lowest scores are healthiest

*PSEQ – Pain Self-Efficacy Questionnaire –* highest scores are healthiest

*PCS: Pain Catastrophisation Scale*- lowest scores are healthiest

*Tampa Scale for Kinesiophobia –* lowest scores are healthiest

